# Variation in Severity-Adjusted Resource use and Outcome for Neurosurgical Emergencies in the Intensive Care Unit

**DOI:** 10.1007/s12028-023-01723-3

**Published:** 2023-04-26

**Authors:** Rahul Raj, André Moser, Joel Starkopf, Matti Reinikainen, Tero Varpula, Stephan M. Jakob, Jukka Takala

**Affiliations:** 1 Department of Neurosurgery, Helsinki University Hospital and University of Helsinki, Helsinki, Finland; 2 CTU Bern, University of Bern, Bern, Switzerland; 3 Anaesthesiology and Intensive Care Clinic, University of Tartu and Tartu University Hospital, Tartu, Estonia; 4 Department of Anesthesiology and Intensive Care, Kuopio University Hospital and University of Eastern Finland, Kuopio, Finland; 5 Division of Intensive Care, University of Helsinki and Helsinki University Hospital, Helsinki, Finland; 6 Department of Intensive Care Medicine, Bern University Hospital, University of Bern, Bern, Switzerland

**Keywords:** Intensive care, Critical care, Traumatic brain injury, Subarachnoid hemorrhage, Intracerebral hemorrhage, Costs

## Abstract

**Background:**

The correlation between the standardized resource use ratio (SRUR) and standardized hospital mortality ratio (SMR) for neurosurgical emergencies is not known. We studied SRUR and SMR and the factors affecting these in patients with traumatic brain injury (TBI), nontraumatic intracerebral hemorrhage (ICH), and subarachnoid hemorrhage (SAH).

**Methods:**

We extracted data of patients treated in six university hospitals in three countries (2015–2017). Resource use was measured as SRUR based on purchasing power parity-adjusted direct costs and either intensive care unit (ICU) length of stay (costSRUR_length of stay_) or daily Therapeutic Intervention Scoring System scores (costSRUR_Therapeutic Intervention Scoring System_). Five a priori defined variables reflecting differences in structure and organization between the ICUs were used as explanatory variables in bivariable models, separately for the included neurosurgical diseases.

**Results:**

Out of 28,363 emergency patients treated in six ICUs, 6,162 patients (22%) were admitted with a neurosurgical emergency (41% nontraumatic ICH, 23% SAH, 13% multitrauma TBI, and 23% isolated TBI). The mean costs for neurosurgical admissions were higher than for nonneurosurgical admissions, and the neurosurgical admissions corresponded to 23.6–26.0% of all direct costs related to ICU emergency admissions. A higher physician-to-bed ratio was associated with lower SMRs in the nonneurosurgical admissions but not in the neurosurgical admissions. In patients with nontraumatic ICH, lower costSRURs were associated with higher SMRs. In the bivariable models, independent organization of an ICU was associated with lower costSRURs in patients with nontraumatic ICH and isolated/multitrauma TBI but with higher SMRs in patients with nontraumatic ICH. A higher physician-to-bed ratio was associated with higher costSRURs for patients with SAH. Larger units had higher SMRs for patients with nontraumatic ICH and isolated TBI. None of the ICU-related factors were associated with costSRURs in nonneurosurgical emergency admissions.

**Conclusions:**

Neurosurgical emergencies constitute a major proportion of all emergency ICU admissions. A lower SRUR was associated with higher SMR in patients with nontraumatic ICH but not for the other diagnoses. Different organizational and structural factors seemed to affect resource use for the neurosurgical patients compared with nonneurosurgical patients. This emphasizes the importance of case-mix adjustment when benchmarking resource use and outcomes.

**Supplementary Information:**

The online version contains supplementary material available at 10.1007/s12028-023-01723-3.

## Introduction

Intensive care demands extensive resources to prevent death and disability. Neurosurgical admissions constitute of up to 20% of all intensive care unit (ICU) admissions to tertiary ICUs [1]. We recently showed that patients undergoing neurosurgery have a relevant impact on ICU resource use: the direct costs of admission and in-hospital mortality are lower than those of the general ICU patient population. However, many patients in the ICU with neurosurgical emergencies do not undergo surgery, for example, those with spontaneous intracranial hemorrhage (ICH), subarachnoid hemorrhage (SAH), and traumatic brain injury (TBI). Mortality rates of neurosurgical emergencies treated in the ICU are high, up to 30–40% after severe TBI or spontaneous ICH [2, 3].

The benchmarking of ICU performance requires the joint assessment of ICU-related outcomes and resource use to provide a health care system relevant summary. Yet, the impact of neurosurgical emergencies on ICU resource use is not known. We recently showed a decrease in standardized mortality ratios (SMRs) over time in all ICU-treated patients without a concomitant increase in severity-adjusted resource use to achieve hospital survivors (standardized resource use ratio [SRUR]) [4]. However, the association between SMR and SRUR, and the factors affecting these, in different neurosurgical emergencies treated in the ICU and their contribution to overall resource use and outcomes of emergency ICU admissions are unknown.

Thus, we studied the SRUR and SMR, and the factors affecting these, of typical neurosurgical emergencies (nontraumatic ICH, SAH, isolated TBI, and multitrauma TBI) in comparison to the SRUR and SMR of nonneurosurgical emergency ICU admissions in ICUs with in-house neurosurgical service. We hypothesized that both SRUR and SMR of neurosurgical emergencies may differ from nonneurosurgical emergencies, the neurosurgical emergencies being more resource-demanding with higher mortality rates. Our findings might impact future ICU resource planning and might improve resource allocation for patients admitted to the ICU.

## Material and Methods

We used anonymized data from 2015 to 2017 from a benchmarking database including university hospital ICUs providing neurosurgical care [5]. The study protocol, database contents and data management process were approved by the National Institute of Health and Welfare, Finland (Decision THL/1524/5.05.00/2017 and THL/1173/05.00/2018). According to regulations in Finland, Estonia, and Switzerland, no ethics committee approval was needed. We adhered to the Strengthening the Reporting of Observational studies in Epidemiology recommendations [6].

The Finnish Intensive Care Consortium (FICC) consists of all adult ICUs in Finland except one neurosurgical ICU (*n* = 21), one university hospital ICU in Estonia (100% of university ICU admissions in Estonia,) and one university hospital ICU in Switzerland (33% of all university hospital ICU admissions in Switzerland). One FICC ICU in Finland declined participation, and one Finnish cardiac hospital ICU was excluded. Furthermore, four Finnish nonuniversity ICUs did not have direct costs available and were excluded. Thus, all six university ICUs with in-house neurosurgical services were included in this study. The units are numbered U2, U3, U4, U5, U7, and U8, according to the original definition [4].

Data on diagnosis, severity of illness, care interventions, and physiologic, administrative, and hospital outcome data from all ICU admissions were extracted from electronic patient records (manually from paper in Estonia) into a validation software. Using logical rules, median filtering, and graphic displays to ensure data quality, each admission was validated by trained ICU nurse data managers or intensivists before transfer to the FICC database.

### Study Population

**Fig. 1:**
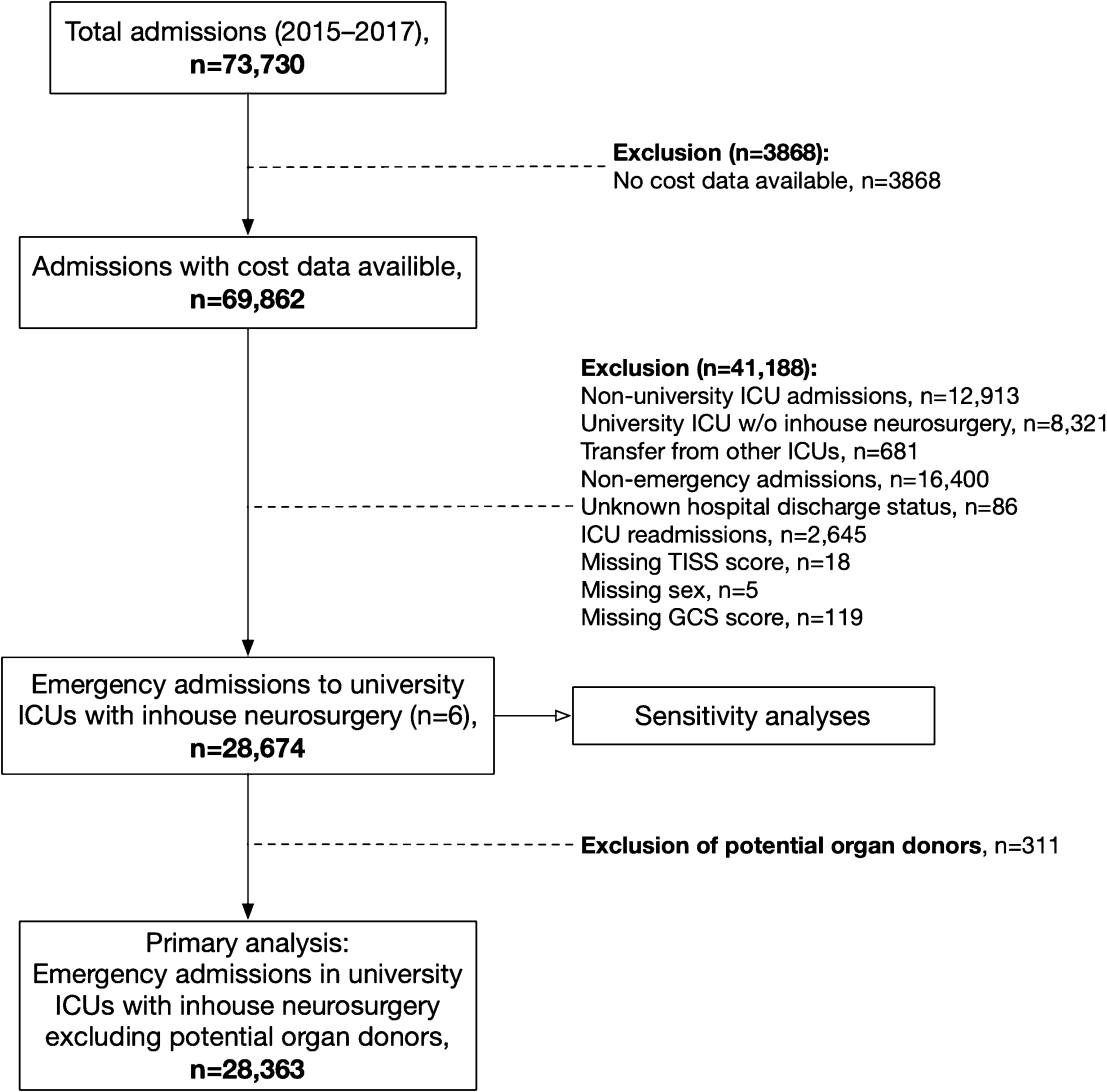
Flow chart showing study population and exclusions. ICU, intensive care unit, GCS, Glasgow Coma Scale, TISS, Therapeutic Intervention Scoring System

We used admissions between 2015 and 2017 from ICUs with cost data available [4]. We included patients with emergency admissions to ICUs with full in-house neurosurgical service (six university hospital ICUs: four in Finland and one each in Estonia and Switzerland). We considered as neurosurgical emergencies patients admitted for a nontraumatic ICH, SAH, isolated TBI, and multitrauma TBI. We excluded transfers from other ICUs; nonemergency admissions; those with an unknown hospital discharge status; readmissions; those with a missing Therapeutic Intervention Scoring System (TISS)-76 score, missing sex, or missing Glasgow Coma Scale (GCS) score; and those admitted as potential organ donors (Fig. 1).

### Resource Use

We assessed resource use using ICU length of stay (LOS), daily collected extended TISS scores (TISS-76 scores, including 17 additional items (Supplementary Information eTable 1), referred to as “TISS”), and direct ICU costs (salaries, drugs, fluids, disposables) as previously described [4]. To include physicians’ salaries allocated to other budgets or simultaneously covering other services, physician staff organization, rotations, and in-house and on-call coverage were clarified with each ICU leader, and costs were allocated based on consensus time estimates [4].

### Calculation of costSRUR

Calculation of costSRUR has been described in detail, including a practical example [4]. Briefly, all yearly ICU admissions were stratified according to Simplified Acute Physiology Score II (SAPS II) scores (0–9, 10–19,…, 80–89, > 90) [7]. The expected resource use per survivor in each SAPS II stratum was the sum of LOS days or TISS scores divided by the number of hospital survivors. For each ICU, the number of survivors multiplied by the expected resource use per survivor in each stratum was calculated, and the sum of all strata was the expected resource use for each ICU.

In the present study, the observed and expected resource uses were calculated specifically for the subgroups of interest (nontraumatic ICH, SAH, isolated TBI and multitrauma TBI, and nonneurosurgical emergencies).

First, the proportion of all ICU direct costs was assigned to the study cohort in each ICU as equal to the study cohort’s proportion of the ICU’s total LOS and TISS, respectively. The sum of these costs in all ICUs provided the total direct costs of the study cohort.

Second, we applied in the diagnostic subgroups the same procedure as described above to calculate the expected resource use per survivor after assigning the proportion of total resource use (as direct costs, LOS, and TISS) to each diagnostic subgroup: the expected costs to produce a hospital survivor in each SAPS II stratum were calculated as (expected resource use LOS/TISS per survivor) × (mean cost of LOS/TISS). For each ICU, the sum of expected direct costs in all strata (number of survivors × expected costs to produce a hospital survivor) was the expected total direct cost [4].

The cost-based SRURs for each ICU were then calculated as observed/expected total direct costs based on LOS (costSRUR_LOS_) and direct costs based on TISS (costSRUR_TISS_).

We used a fixed exchange rate of 1.09 Swiss franc to 1.00 euro (EUR), without inflation adjustment, and we adjusted the costs for purchasing power parity [4, 8].

### Calculation of SMR

We calculated SMR for all ICUs as observed/predicted hospital mortality. We calculated the predicted hospital mortality using a recent prediction model customized for FICC [9]. Briefly, the risk model includes patient age, a modified SAPS II score (without age and admission type), patient premorbid functional status, and APACHE III diagnosis and admission type (emergency for all). GCS score was defined according to the SAPS II criteria as the worst score during the first 24 h in the ICU or the last score preceding sedation for intubated/sedated patients.

### Statistical Analysis

We followed a similar analysis strategy as previously described [4]. We described the study population by frequencies (*n*), percentages (%), and medians and interquartile ranges. We used box plots to describe SMR and costSRUR measures across the different diagnostic groups. We used gamma distributed regression models to investigate a priori defined ICU-related factors (total number of ICU beds, full-time equivalent [FTE] physicians-to-bed ratio, organization type [independent organization vs. part of another department], median SAPS and SMR) associated with costSRUR_LOS_ and costSRUR_TISS_. We investigated the ICU-related factors' association with SMR and costSRURs on the ICU level for each diagnosis group separately. To avoid overfitting of the model, we reported only the bivariable results [10].

In the primary analysis, we excluded patients admitted as potential organ donors from both resources and outcomes [9]. We conducted three sensitivity analyses in which we (1) included used resources but excluded the number of survivors for patients admitted as potential organ donors and (2) included used resources and the number of survivors for patients admitted as potential organ donors.

Continuous variables were standardized (centered and expressed per 1 standard deviation increase) and relative risk estimates reported with 95% confidence intervals. All analyses were performed in R version 4.1.2 (R Team Core. R: A language and environment for statistical computing; R Foundation for Statistical Computing, Vienna, Austria).

## Results

### Patient Characteristics

Of all 28,674 patients treated in a university hospital with in-house neurosurgical services not fulfilling one of the other exclusion criteria (eTable 2), 311 were admitted as potential organ donors (Fig. 1). Of the 28,363 remaining patients, 6,162 (22%) were admitted with a neurosurgical disease. Patients with a neurosurgical diagnosis had lower GCS scores, had lower SAPS II scores, were more frequently admitted after surgery, more often had a normal premorbid functional status, and had higher extended TISS scores, longer ICU stays, and a lower hospital mortality rate (9.3% vs. 13.0%) than patients with a nonneurosurgical diagnosis (Table 1). Of the neurosurgical diagnoses, nontraumatic ICH was the most common diagnosis (41% of all admissions), followed by isolated TBI (24%), SAH (23%), and multitrauma TBI (13%).

**Table 1:** Study population with neurosurgical diagnoses and nonneurosurgical diagnoses

Characteristic	Neurosurgical diagnoses (*n* = 6,162)	All other diagnoses (*n* = 22,201)
Age	62 (48–73)	63 (49–74)
Female sex	2,382 (39)	8,551 (39)
GCS score	13.0 (7.0–14.0)	14.0 (11.0–15.0)
SAPS II score	31 (22–45)	36 (25–49)
Modified SAPS II score	15 (10–20)	21 (13–29)
Diagnostic group		
All other diagnoses	–	22,201 (100)
Nontraumatic intracranial hemorrhage	2,514 (41)	–
SAH	1,394 (23)	–
Multitrauma TBI	814 (13)	–
Isolated TBI	1,440 (23)	–
Operative admission	2,272 (37)	5,530 (25)
Premorbid functional status		
Normal	5,098 (83)	15,498 (70)
Light limitation	754 (12)	4,101 (18)
Moderate limitation	235 (3.8)	1,921 (8.7)
Severe limitation	75 (1.2)	681 (3.1)
Hospital mortality	574 (9.3)	2,788 (13.0)
Extended TISS-76 score, total	70 (36–176)	59 (33–128)
Length of ICU stay	1.9 (0.9–4.9)	1.5 (0.8–3.1)

Continuous variables reported as median (IQR) and categorical variables as *n* (%). The modified SAPS II score is defined as the SAPS II score without age and admission type

*GCS* Glasgow Coma Scale, *ICU* intensive care unit, *IQR* interquartile range, SAH, subarachnoid hemorrhage, *SAPS* II, Simplified Acute Physiology Score II, *TBI* traumatic brain injury, *TISS* therapeutic intervention scoring system

Patients with multitrauma TBI were the youngest patient group, and patients with nontraumatic ICH were the oldest patient group. Women were overrepresented in the SAH group. SAPS II score distribution was similar between the diagnostic groups. Patients with SAH and multitrauma TBI had higher TISS scores and longer LOSs than the other groups (Table 2).

**Table 2:** Patient characteristics according to neurosurgical diagnosis

Characteristic	Nontraumatic ICH (*n* = 2514)	SAH (*n* = 1394)	Multitrauma TBI (*n* = 814)	Isolated TBI (*n* = 1440)
Age	68 (57–77)	58 (48–67)	49 (28–67)	59 (39–71)
Female sex	935 (37)	817 (59)	226 (28)	404 (28)
GCS score	12.0 (7.0–14.0)	13.0 (6.0–15.0)	13.0 (7.0–14.0)	13.0 (7.0–14.0)
SAPS II score	34 (26–47)	28 (20–46)	31 (20–44)	29 (20–42)
Modified SAPS II score	15 (10–20)	14 (10–20)	17 (12–22)	13 (10–19)
Operative admission	1,243 (49)	384 (28)	205 (25)	440 (31)
Premorbid functional status				
Normal	2,011 (80)	1,207 (87)	747 (92)	1,133 (79)
Light limitation	327 (13)	152 (11)	46 (5.7)	229 (16)
Moderate limitation	132 (5.3)	22 (1.6)	18 (2.2)	63 (4.4)
Severe limitation	44 (1.8)	13 (0.9)	3 (0.4)	15 (1.0)
Hospital mortality	281 (11)	148 (11)	53 (6.5)	92 (6.4)
Extended TISS-76 score, total	61 (30–136)	121 (53–319)	82 (40–228)	54 (30–120)
Length of ICU stay	1.7 (0.8–3.8)	3.4 (1.6–9.2)	2.3 (1.0–6.5)	1.5 (0.8–3.4)

Continuous variables reported as median (IQR) and categorical variables as *n* (%). The modified SAPS II score is defined as the SAPS II score without age and admission type

*GCS* Glasgow Coma Scale, *ICH* intracranial hemorrhage, *ICU* intensive care unit, *IQR* interquartile range, *SAH* subarachnoid hemorrhage, *SAPS* II, Simplified Acute Physiology Score II, *TBI* traumatic brain injury, *TISS* Therapeutic Intervention Scoring System

There were some differences in patient characteristics between units (eTable 3). Median age varied between 59 and 67 years, median GCS score between 5 and 13, and normal preadmission premorbid functional status between 56 and 94%. The proportion of neurosurgical-to-total admissions varied between 19 and 23%. Hospital mortality increased with a decreasing GCS score, with some notable differences in-hospital mortality rate among patients with GCS ≤ 8 across units (eFig. 1).

### Severity-Adjusted Mortality and Resource Use to Achieve Hospital Survivors

**Fig. 2:**
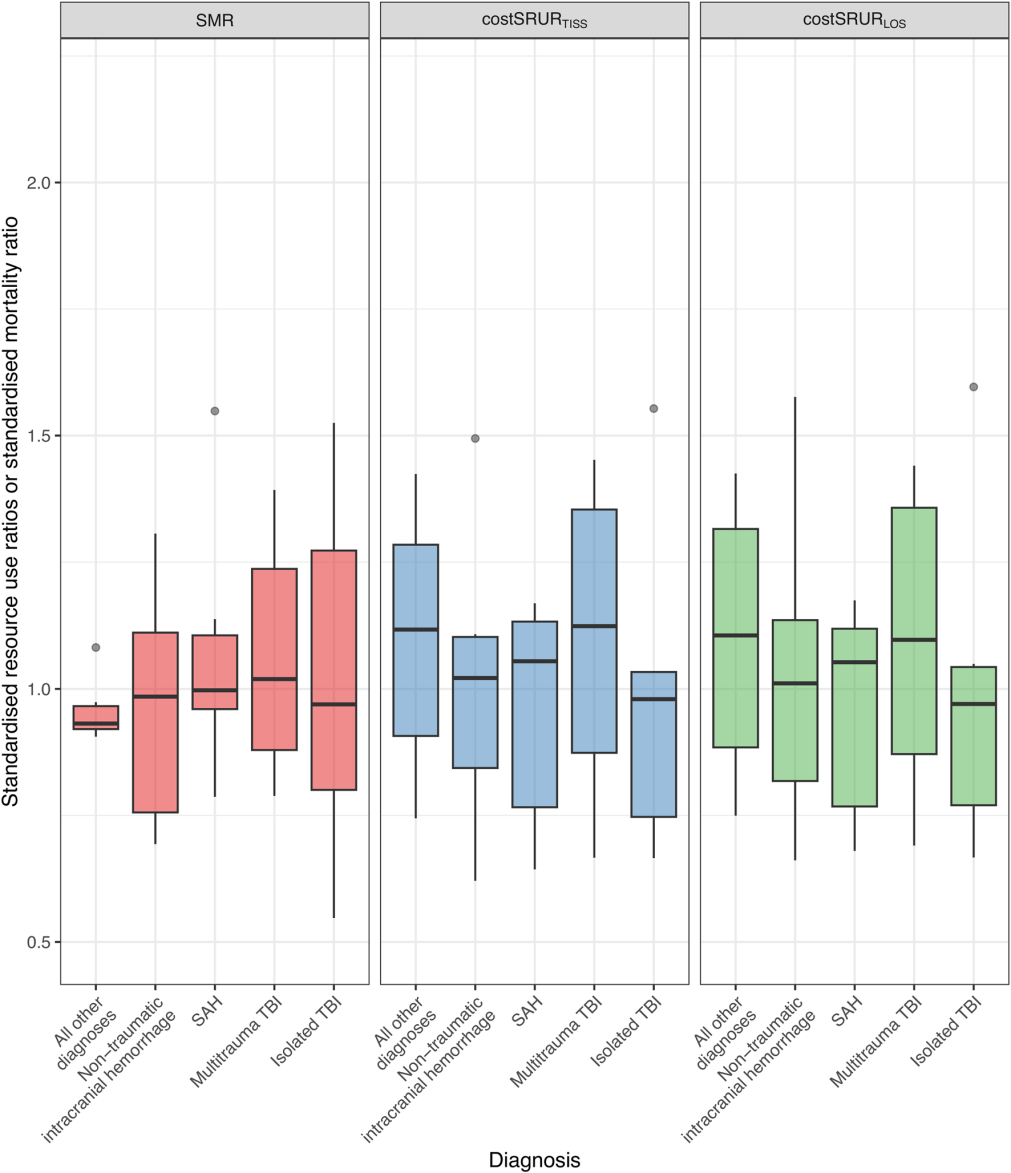
Differences in standardized mortality ratio (SMR) and standardized resource use ratios (costSRUR_TISS_, costSRUR_LOS_) between the neurosurgical diagnoses. Box plots show the median, the first and third quartiles, and whiskers defined by 1.5 times the interquartile range. Values are reported in eTable 4. costSRUR_LOS_, xxx, costSRUR_TISS_, xxx, SAH, subarachnoid hemorrhage, TBI, traumatic brain injury

A comparison between SMR, costSRUR_TISS_, and costSRUR_LOS_ is shown in Fig. 2 and eTable 4. We found no significant differences in SMR or costSRUR measures between the neurosurgical and nonneurosurgical diagnoses or within the neurosurgical diagnoses.

The annual direct costs for all ICU emergency admissions varied from 67.1 million EUR to 70.4 million EUR (TISS-based cost separation) and from 70.0 million EUR to 72.9 million EUR (LOS-based cost separation). The corresponding direct costs for neurosurgical emergency admissions represented 23.6–23.7% (TISS-based) and 25.7–26.0% of direct costs for all emergency admissions.

The mean costs per patient with neurosurgical diagnoses were highest for patients with SAH (12,466 EUR) and patients with multitrauma TBI (11,115 EUR) and lowest for patients with isolated TBI (6,978 EUR). The mean cost per patient with other diagnoses was 6,749 EUR (eTable 5). The mean costs per LOS day did not notably differ across groups (1,981–1,988 EUR per LOS day, whereas the costs per TISS score were higher in patients with neurosurgical diagnoses (50.6–54.5 EUR per TISS score vs. 48.2 EUR per TISS score in patients with other diagnoses).

The direct costs per survivor increased exponentially with increasing SAPS II category for all diagnoses, except for patients with SAH, in which the cost per survivor in the highest SAPS II categories, containing 1% of admissions, decreased (eTable 6, eFig. 2).

### Associations Between Severity-Adjusted Mortality, Resource Use to Achieve Hospital Survivors, and ICU-Related Factors

**Fig. 3:**
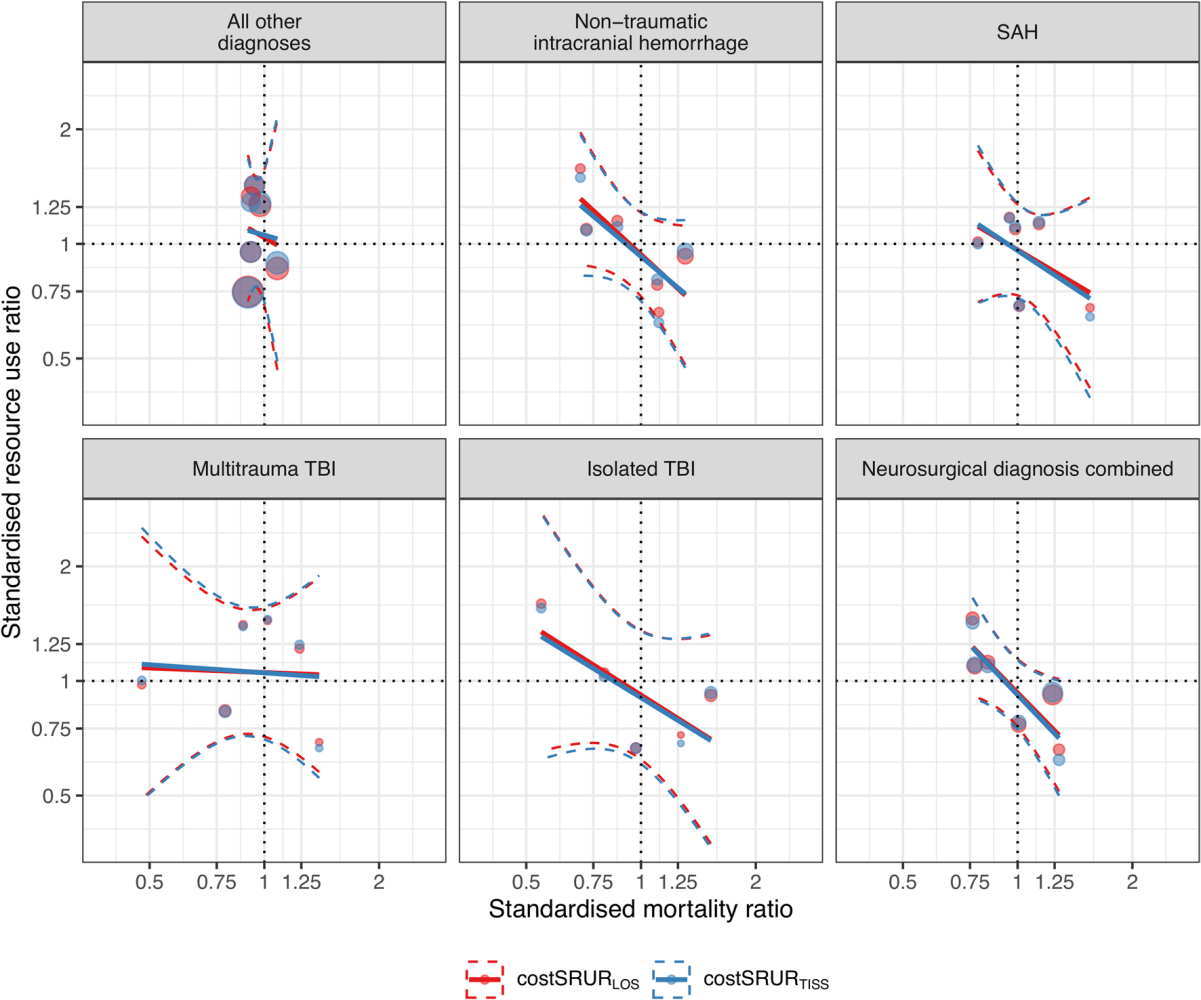
Standardized resource use ratios (costSRUR_LOS_, costSRUR_TISS_) in relation to the standardized mortality ratio (SMR) for all other nonneurosurgical patients and the included neurosurgical diagnoses. Filled circles: an ICU, circle size is proportional to the number of ICU admissions. Solid lines: Gaussian linear regression lines. Dashed lines: their 95% confidence intervals (slope estimates in eTable 7). Dotted horizontal and vertical lines: costSRUR = 1 and SMR = 1. A significant association between SMR and costSRUR_LOS_/costSRUR_TISS_ was found in the combined neurosurgical diagnoses group (bottom right) and in patients with nontraumatic ICH (upper mid). costSRUR_LOS_, xxx, costSRUR_TISS_, xxx, ICH, intracranial hemorrhage, ICU, intensive care unit, SAH, subarachnoid hemorrhage, TBI, traumatic brain injury

We found an association between higher costSRUR_TISS_ and costSRUR_LOS_ and lower SMR when combining all neurosurgical diagnoses (Fig. 3, eTable 7). Separating the neurosurgical diagnoses, the association between higher costSRUR_TISS_ and costSRUR_LOS_ and lower SMR was apparent only for patients with nontraumatic ICH.

**Fig. 4:**
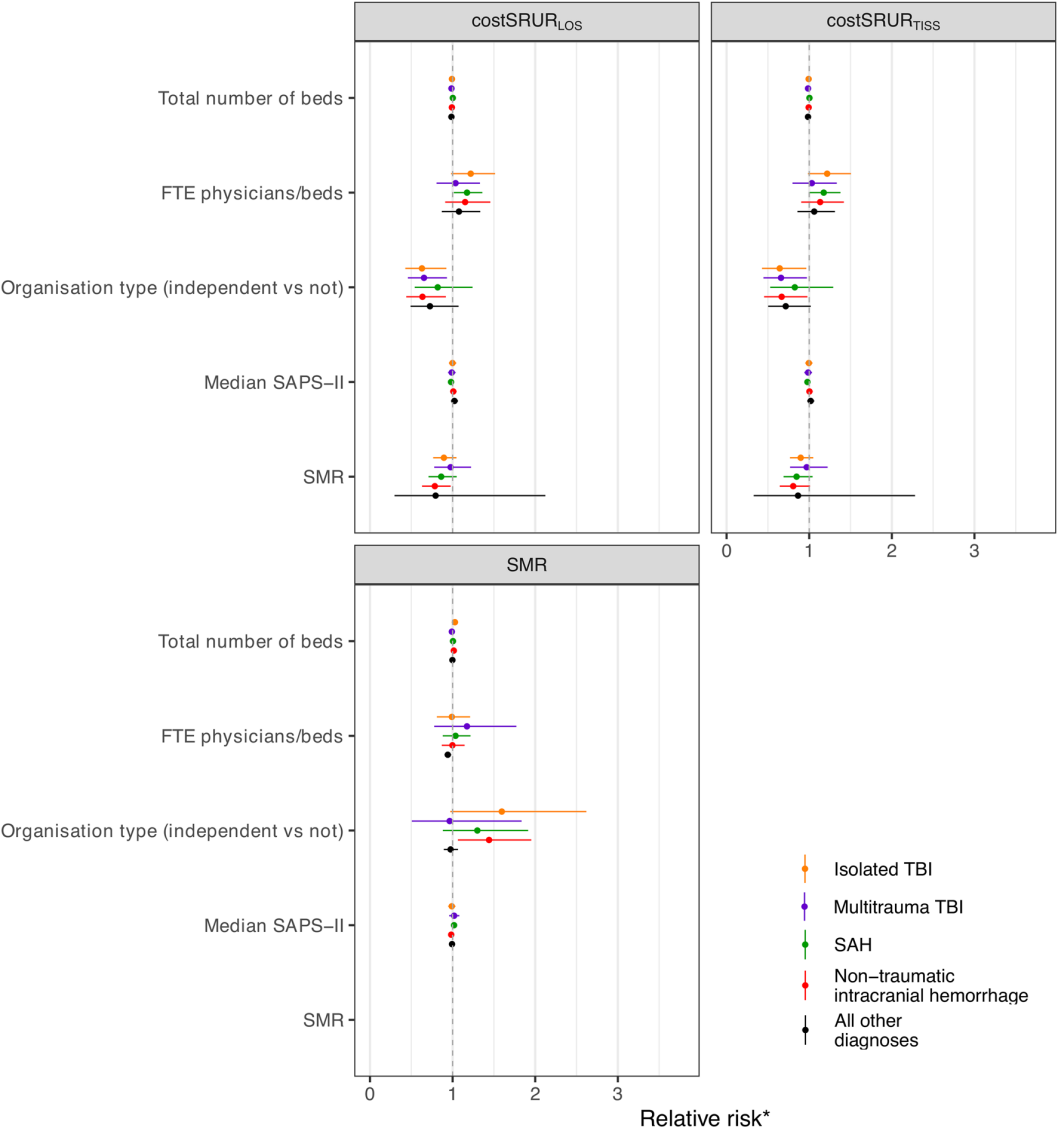
Bivariable analyses of variables associated with standardized resource utilization ratios (costSRUR_TISS_, costSRUR_LOS_) and standardized mortality ratio (SMR). Values are reported in eTable 8. costSRUR_LOS_, xxx, costSRUR_TISS_, xxx, FTE, full-time equivalent, ICH, intracranial hemorrhage, ICU, intensive care unit, SAH, subarachnoid hemorrhage, SAPS-II, Simplified Acute Physiology Score II, TBI, traumatic brain injury

A higher FTE physician-to-bed ratio was associated with higher costSRUR_LOS_ and costSRUR_TISS_ in patients with SAH (Fig. 4, eTable 8). A higher FTE physician-to-bed ratio was associated with a lower SMR for the nonneurosurgical diagnoses but not for any of the neurosurgical diagnoses.

An independent organization type was associated with lower costSRURs (costSRUR_LOS_, costSRUR_TISS_) in patients with nontraumatic ICH, isolated TBI, and multitrauma TBI. For patients with nontraumatic ICH, an independent organization type was associated with a higher SMR in the primary and both sensitivity analyses. Furthermore, a higher SMR was associated with a lower costSRUR_LOS_ in patients with nontraumatic ICH.

There was an association between a higher total number of beds and a higher SMR in patients with nontraumatic ICH and isolated TBI. The total number of beds was not associated with either of the SRUR measures.

A higher median SAPS II was associated with lower costSRURs in patients with SAH.

All bivariable associations remained statistically significant in both sensitivity analyses, with the exception of the association between organization type and lower costSRUR_TISS_ for patients with nontraumatic ICH and the association between a higher FTE physician-to-bed ratio and costSRUR_TISS_ for patients with SAH in the sensitivity analysis in which resources used for potential organ donors were included, but the number of survivors of the potential organ donors were excluded.

## Discussion

Neurosurgical diseases constituted 22% of all emergency admissions and approximately 25% of the direct costs of all emergency admissions in university hospital ICUs with in-house neurosurgical service. Compared with other ICU emergency admissions, the neurosurgical emergency admissions more often had a normal premorbid functional status and surgery before ICU admission and had lower SAPS II and GCS scores, longer LOS and higher TISS, and lower hospital mortality. The costs per admission and per TISS were higher in neurosurgical emergency admissions than in other emergency admissions, but there were relevant differences between the neurosurgical diagnostic groups. The costs per admission were highest in patients with SAH and lowest in patients with isolated TBI. Despite these differences, the SMRs and the severity-adjusted resources needed to achieve survivors, measured by costSRURs, were similar to those of nonneurosurgical emergencies, probably due to differences in case-mix severity. This finding further emphasizes the need to include severity adjustment in the evaluation of resource use and outcomes.

Despite this, we found more than twofold variation between the individual ICUs in the severity-adjusted resource use to achieve survivors (costSRURs) in all diagnostic groups, including other than neurosurgical emergencies. Including all neurosurgical diagnoses, we found a statistically significant association between higher costSRURs and lower SMR, suggesting that the higher severity-adjusted costs to achieve survivors were associated with improved outcomes. However, separating the individual neurosurgical diagnoses, a higher costSRUR was only associated with a lower SMR in patients with nontraumatic ICH. A similar trend was observed for patients with isolated TBI and SAH, although it was not statistically significant (eTable 7). Thus, these findings are in line with our previous findings, in which we did not find any consistent associations between costSRUR and SMR [1, 4]. However, the large variation in SMRs between ICUs in the neurosurgical diseases, most prominent in the TBI groups, was unexpected given the much smaller variation of SMRs in other emergency ICU admissions [4].

Some previous studies suggest that specialized neurosurgical ICUs may offer outcome benefits, although the results are not conclusive [11–14]. Our sample size including only six ICUs did not allow multivariable analyses on structural and organizational variables that may be associated with SRURs and SMR. We performed a limited number of prespecified bivariable analyses. Only in patients with nontraumatic ICH were lower costSRURs associated with a higher SMR, suggesting that lower resource use could lead to higher mortality in this patient group (eTables 7 and 8). A higher FTE physician-to-bed ratio was associated with a lower SMR for patients with nonneurosurgical admissions but not for those with neurosurgical admissions. This may suggest that the initial prognosis and the neurosurgical treatment rather than intensive care may be more relevant for outcome for neurosurgical emergencies. Due to the bivariable analysis, this association should be considered with caution.

We observed that an independent organization type was associated with lower costSRURs in patients with nontraumatic ICH, isolated TBI, and multitrauma TBI. This was contrasted by an association between independent organization type and a higher SMR for patients with nontraumatic ICH and by an association between a higher total number of beds and a higher SMR in patients with nontraumatic ICH and isolated TBI. These bivariable associations must be interpreted with caution due to several confounding variables and differences in the case-mix model. For example, four of the six ICUs had an independent organization, and all six had more than 20 beds. A well-known confounder for SMR is the assessment of the GCS score in patients who are often sedated. It is possible that this is relevant for our study, as our study demonstrates wide variability between individual ICUs in mortality of patients with the worst GCS score. For example, the mortality of patients with a GCS score 3–5 was lower in both nonindependent units than the mortality of patients with a GCS score 3–5 in the four independent units (26–31% vs. 35–57%, mean for all units 42%; eTable 2), which may falsely reduce the SMRs of individual units and not be detected in bivariable analyses. Nevertheless, the impact of this and such organizational factors, such as the impact of smaller dedicated neurosurgical ICUs, needs to be further studied [15, 16].

### Limitations

There are some limitations that should be acknowledged. First, we used a SAPS II–based case-mix model [9] that may not be optimal for neurosurgical diseases. It is possible that SAPS II overestimates the injury severity in neurosurgical patients who may have an initially lowered but correctable GCS score. For example, up to 50% of patients with SAH develop acute hydrocephalus requiring external ventricular drainage, which can temporarily lower the noted GCS score [17], or a patient with an unknown GCS score before starting sedation was given a GCS score reflecting sedation. However, because the FICC database does not contain data regarding EVD placement or the exact time-point of GCS assessment, we were unable to control for this. Second, due to the limited number of patients in the separate groups, we used bivariable modeling, which may not capture interactions between the variables. Third, most of the confidence intervals that do not include 1.0 are very close to it, and their relevance needs to be interpreted cautiously. Fourth, intercountry variations in organ donation policies may affect our results. Among the included three countries, the number of organ donations is the highest among Finnish centers, being in 2021 on average 22 of 1,000,000 compared with 16 of 1,000,000 in Estonia and 19 of 1,000,000 in Switzerland [18]. Because most of the included patients come from Finnish centers, this might possibly affect our results. Importantly, patients admitted as potential organ donors have an expected mortality of 100%, which is, in many instances, higher than the expected mortality according to their SAPS II score. Still, the sensitivity analyses did not alter our results, and thus the impact on variations in organ donations is probably negligible. Fifth, although it is an important measure for ICU performance, hospital mortality may not adequately reflect outcomes after ICU-treated neurosurgical emergencies, in which long-term neurological outcomes are of more relevance. For example, in a previous study, it was shown that patients with nontraumatic ICH had the poorest prognosis and highest costs per independent survivor out of several neurosurgical emergencies [19].

## Conclusions

We conclude that neurosurgical emergencies constitute a major proportion of all emergency ICU admissions. Despite lower hospital mortality, the costs of care are higher and the LOS is longer than other emergency admissions. When adjusted for severity, the resources needed to achieve survivors and hospital mortality in patients admitted to the ICU with neurosurgical emergencies are similar to those of nonneurosurgical emergencies. Our results emphasize the need to include severity adjustment in the evaluation of resource use and outcomes.

### Supplementary Information

Below is the link to the electronic supplementary material.

Supplementary file1 (PDF 1444 KB)
