# Supplementary material for: Variation in Severity-Adjusted Resource use and Outcome for Neurosurgical Emergencies in the Intensive Care Unit
Source: Neurocrit Care. 2023 Apr 26;40(1):251–61. doi: 10.1007/s12028-023-01723-3 (PMC10861740; doi:10.1007/s12028-023-01723-3)

Variation in severity-adjusted resource use and outcome for neurosurgical emergencies in the intensive care unit

Raj R, Moser A, Starkopf J, Reinikainen M Varpula T, Jakob SM*, Takala J*

Table of Content

eTable 1: Extended TISS variables and their description.......... 2
eTable 2: Overall study population 3
eTable 3: Study population with neurosurgical diagnoses according to unit.......... 4
eTable 4: Numeric values for the SMR and SRUR measure comparison between the different diagnostic groups showed in Figure 2 5
eTable 5: Variation across units and years 6
eTable 6: Variation in directs costs per survivor (LOS and TISS) according to SAPS II strata. Stratified across units, SAPS categories and years. 7
eTable 7: Slope estimates for the relation between costSRURs and SMR across diagnostic groups for Figure 3.......... 9
eTable 8: Association of ICU-related factors with costSRURs and SMR 10
eFigure 1: The relationship between GCS score and hospital mortality across units 13
eFigure 2: Direct TISS and LOS-based cost per survivor by SAPS II strata 14

eTable 1: Extended TISS variables and their description

Variable	Description	Score
Dangerously or continuously agitated patient	Patient is dangerous to her/himself or to the staff, or anxiety is a noticeable problem affecting the care of the patient.	4
Demanding hygiene actions	Hygiene actions are required more than once per shift.	3
Demanding rehabilitation	More than usual rehabilitation: needs two or more nurses at the same time.	3
Demanding communication and support to family	Communication with and support of the patient’s relatives for more than 1 hour per shift.	3
Donor patient care	Support of a donor patient (does not require diagnosed brain death).	4
EEG examination/monitoring	EEG examination and monitoring performed by ICU staff or others.	3
Extracorporeal Membrane Oxygenation	ECMO (Extracorporeal Membrane Oxygenation) in care of pulmonary or circulatory dysfunction.	4
Investigation outside of the ICU	Interventions outside ICU (for example CT scan).	4
Isolated patient	Clean or contaminated isolation: applies if patient is treated in separate room because of danger of infection.	4
Medication into the epidural catheter	Medication into the epidural catheter.	2
Molecular Adsorbent Recirculating System	MARS treatment in care of liver dysfunction.	4
Negative pressure wound treatment (NPWT) - Demanding wound care	Demanding wound care (for example fitting or changing NPWT).	2
Prone position	Prone position in care of respiratory failure.	4
Showering mechanically ventilated patient	Showering mechanically ventilated patient.	4
Thrombolytic therapy	Thrombolysis therapy in the ICU.	3
Ultrasound	Ultrasound examination in the ICU. If performed outside ICU "Investigation outside of the ICU " should be marked. Using ultrasound for canulation is excluded	2
Wound care for burn patient	Demanding wound care for burn patient: needs two or more nurses at the same time for more than one hour.	4

eTable 2: Overall study population

Characteristic	N = 28,363
**Age**	63 (49, 73)
**Female**	10,933 (39%)
**GCS score**	14.0 (10.0, 15.0)
**SAPS II score**	35 (25, 48)
**Modified SAPS II score**	19 (13, 27)
**Diagnostic group**	
All other diagnoses	22,201 (78%)
Non-traumatic intracranial hemorrhage	2,514 (8.9%)
SAH	1,394 (4.9%)
Multitrauma TBI	814 (2.9%)
Isolated TBI	1,440 (5.1%)
**Operative admission**	7,802 (28%)
**Premorbid functional status**	
Normal	20,596 (73%)
Light limitation	4,855 (17%)
Moderate limitation	2,156 (7.6%)
Severe limitation	756 (2.7%)
**Hospital mortality**	3,362 (12%)
**Extended TISS-76 score, total**	61 (33, 136)
**Length of ICU stay**	1.6 (0.8, 3.6)

Continuous variables reported as median (IQR) and categorical variables as n (%)

The modified SAPS II score is defined as the SAPS II score without age and admission type

Abbreviations: ICU, intensive care unit; GCS, Glasgow Coma Scale; SAPS, Simplified Acute Physiology Score; SAH, subarachnoid hemorrhage; TBI, traumatic brain injury; TISS, Therapeutic Intervention Scoring System

eTable 3: Study population with neurosurgical diagnoses according to unit

Characteristic	U2, N = 589	U3, N = 1,118	U4, N = 909	U5, N = 771	U7, N = 2,432	U8, N = 343
**Age**	60 (46, 70)	59 (43, 68)	61 (47, 69)	60 (48, 70)	67 (52, 78)	57 (45, 68)
**Female**	260 (44%)	431 (39%)	359 (39%)	303 (39%)	905 (37%)	124 (36%)
**GCS score**	10.0 (5.0, 14.0)	13.0 (8.0, 15.0)	13.0 (7.0, 15.0)	10.0 (5.0, 14.0)	13.0 (9.0, 14.0)	5.0 (3.0, 14.0)
**SAPS II score**	36 (23, 52)	29 (20, 43)	28 (20, 41)	35 (23, 51)	31 (24, 41)	48 (33, 62)
**Modified SAPS II score**	16 (11, 20)	14 (11, 20)	13 (10, 20)	16 (10, 20)	13 (10, 19)	22 (16, 28)
**Diagnostic group**						
All other diagnoses	0 (0%)	0 (0%)	0 (0%)	0 (0%)	0 (0%)	0 (0%)
Non-traumatic intracranial hemorrhage	172 (29%)	359 (32%)	309 (34%)	285 (37%)	1,210 (50%)	179 (52%)
SAH	206 (35%)	298 (27%)	260 (29%)	198 (26%)	349 (14%)	83 (24%)
Multitrauma TBI	61 (10%)	123 (11%)	84 (9.2%)	86 (11%)	415 (17%)	45 (13%)
Isolated TBI	150 (25%)	338 (30%)	256 (28%)	202 (26%)	458 (19%)	36 (10%)
**Operative admission**	240 (41%)	356 (32%)	259 (28%)	365 (47%)	815 (34%)	237 (69%)
**Premorbid functional status**						
Normal	327 (56%)	956 (86%)	594 (65%)	659 (85%)	2,275 (94%)	287 (84%)
Light limitation	212 (36%)	121 (11%)	246 (27%)	43 (5.6%)	95 (3.9%)	37 (11%)
Moderate limitation	41 (7.0%)	21 (1.9%)	50 (5.5%)	63 (8.2%)	50 (2.1%)	10 (2.9%)
Severe limitation	9 (1.5%)	20 (1.8%)	19 (2.1%)	6 (0.8%)	12 (0.5%)	9 (2.6%)
**Hospital mortality**	52 (8.8%)	69 (6.2%)	75 (8.3%)	64 (8.3%)	241 (9.9%)	73 (21%)
**Extended TISS-76 score, total**	125 (56, 326)	78 (45, 153)	70 (38, 157)	108 (47, 251)	45 (24, 120)	126 (58, 272)
**Length of ICU stay**	3.3 (1.2, 8.7)	1.9 (1.0, 4.5)	1.9 (0.9, 4.1)	2.7 (1.2, 6.7)	1.6 (0.8, 3.8)	3.5 (1.5, 7.5)

Continuous variables reported as median (IQR) and categorical variables as n (%)

The modified SAPS II score is defined as the SAPS II score without age and admission type

Abbreviations: ICU, intensive care unit; GCS, Glasgow Coma Scale; SAPS, Simplified Acute Physiology Score; SAH, subarachnoid hemorrhage; TBI, traumatic brain injury; TISS, Therapeutic Intervention Scoring System

eTable 4: Numeric values for the SMR and SRUR measure comparison between the different diagnostic groups showed in Figure 2

Measure	Diagnostic group	Median	25% quartile	75% quartile
SMR	All other diagnoses	0.932	0.921	0.966
SMR	Non-traumatic intracranial hemorrhage	0.985	0.756	1.111
SMR	SAH	0.997	0.960	1.106
SMR	Multitrauma TBI	0.949	0.811	1.182
SMR	Isolated TBI	0.885	0.611	1.197
$costSRUR_{TISS}$	All other diagnoses	1.117	0.907	1.284
$costSRUR_{TISS}$	Non-traumatic intracranial hemorrhage	1.022	0.844	1.103
$costSRUR_{TISS}$	SAH	1.055	0.766	1.133
$costSRUR_{TISS}$	Multitrauma TBI	1.124	0.874	1.354
$costSRUR_{TISS}$	Isolated TBI	0.980	0.747	1.033
$costSRUR_{LOS}$	All other diagnoses	1.106	0.884	1.315
$costSRUR_{LOS}$	Non-traumatic intracranial hemorrhage	1.011	0.818	1.136
$costSRUR_{LOS}$	SAH	1.053	0.768	1.119
$costSRUR_{LOS}$	Multitrauma TBI	1.097	0.871	1.357
$costSRUR_{LOS}$	Isolated TBI	0.970	0.770	1.043

Abbreviation: SMR, Standardized Mortality Rate; TISS, Therapeutic Intervention Scoring System; LOS, Length of Stay; SAH, Subarachnoid Hemorrhage; TBI, Traumatic Brain Injury; SRUR, Standardized Resource Use Rate

eTable 5: Variation across units and years

We calculated the proportion of resources used for the different diagnostic groups as their proportion of total LOS and total TISS and the respective proportion of total directs costs. Because the proportions of subpopulation LOS and TISS of total costs may differ, all cost calculations were done using both proportions.

Tables shows aggregation for mean values, median, range and 25th to 75th percentile based on mean values of individual units and years.

	**All other diagnoses (N=6)**	**Non-traumatic intracranial hemorrhage (N=6)**	**SAH (N=6)**	**Multitrauma TBI (N=6)**	**Isolated TBI (N=6)**	**Overall (N=30)**
**Mean costs, euro**						
Mean	6749	7635	12466	11155	6978	8997
Median	6408	6634	13444	9629	5995	8768
Min	4423	4692	8668	7549	4212	4212
Max	9706	13274	15613	15628	12522	15628
25%	4951	5476	10151	9041	5370	5585
75%	8414	8773	14294	14142	7487	11907
**Mean costs per LOS, euro**						
Mean	1987	1988	1984	1981	1981	1984
Median	2080	2081	2082	2081	2078	2080
Min	1226	1220	1219	1227	1226	1219
Max	2378	2381	2376	2365	2370	2381
25%	1823	1831	1818	1815	1811	1779
75%	2335	2332	2333	2326	2327	2365
**Mean costs per TISS, euro**						
Mean	48.22	53.31	54.46	50.57	53.17	51.95
Median	50.45	56.96	59.00	54.17	57.22	55.16
Min	30.72	31.04	31.77	30.17	31.88	30.17
Max	59.31	65.14	72.20	62.18	63.48	72.20
25%	43.25	49.20	45.74	45.54	49.18	44.02
75%	55.77	61.61	62.38	58.58	61.34	61.40

Aggregation across all admissions for mean values, medians, range and 25th-75th percentile based on mean values of individual ICUs and years

Abbreviation: TISS, Therapeutic Intervention Scoring System; LOS, Length of Stay; SAH, Subarachnoid Hemorrhage; TBI, Traumatic Brain Injury

eTable 6: Variation in directs costs per survivor (LOS and TISS) according to SAPS II strata. Stratified across units, SAPS categories and years.

Diagnostic group	SAPS-II strata	No. of admissions	Direct costs (euro) per survivor (LOS)	Direct costs (euro) per survivor (TISS)
All other diagnoses	0-9	376	Mean 2396, range 1437 to 3768	Mean 1888, range 1303 to 2481
All other diagnoses	10-19	2386	Mean 3293, range 2238 to 11635	Mean 2781, range 1733 to 11791
All other diagnoses	20-29	5141	Mean 3989, range 2538 to 6586	Mean 3553, range 2065 to 5864
All other diagnoses	30-39	5191	Mean 5928, range 4018 to 9413	Mean 5697, range 3651 to 9435
All other diagnoses	40-49	3741	Mean 8647, range 5109 to 13789	Mean 8690, range 5133 to 14109
All other diagnoses	50-59	2441	Mean 11844, range 7721 to 19059	Mean 12426, range 8411 to 19740
All other diagnoses	60-69	1551	Mean 15080, range 9939 to 21293	Mean 16440, range 10923 to 22501
All other diagnoses	70-79	777	Mean 17144, range 8087 to 31594	Mean 19756, range 9268 to 46490
All other diagnoses	80-89	357	Mean 20429, range 13187 to 44300	Mean 25703, range 16423 to 56246
All other diagnoses	90-	240	Mean 30005, range 8095 to 130150	Mean 42134, range 12656 to 164342
Non-traumatic intracranial hemorrhage	0-9	24	Mean 4362, range 1325 to 12858	Mean 3739, range 885 to 9347
Non-traumatic intracranial hemorrhage	10-19	168	Mean 4368, range 1534 to 24491	Mean 3818, range 920 to 22936
Non-traumatic intracranial hemorrhage	20-29	717	Mean 5182, range 1254 to 19852	Mean 4697, range 1362 to 18627
Non-traumatic intracranial hemorrhage	30-39	676	Mean 5967, range 2716 to 15608	Mean 5825, range 2504 to 14743
Non-traumatic intracranial hemorrhage	40-49	395	Mean 10600, range 6225 to 28672	Mean 10746, range 6495 to 28785
Non-traumatic intracranial hemorrhage	50-59	264	Mean 14630, range 6689 to 19287	Mean 15783, range 6544 to 21292
Non-traumatic intracranial hemorrhage	60-69	189	Mean 12597, range 4959 to 31865	Mean 13960, range 5346 to 38430
Non-traumatic intracranial hemorrhage	70-79	66	Mean 11968, range 920 to 35215	Mean 14977, range 1626 to 42270
Non-traumatic intracranial hemorrhage	80-89	14	Mean 15778, range 9148 to 9148	Mean 20930, range 8900 to 8900
Non-traumatic intracranial hemorrhage	90-	1	Mean 1660160, range NA to NA	Mean 2921940, range NA to NA
SAH	0-9	34	Mean 9063, range 1396 to 33346	Mean 7771, range 1363 to 31549
SAH	10-19	243	Mean 5952, range 1336 to 9605	Mean 5212, range 518 to 9203

SAH	20-29	463	Mean 9509, range 6001 to 16884	Mean 8592, range 4569 to 17156
SAH	30-39	209	Mean 17449, range 4810 to 30682	Mean 17331, range 3612 to 30818
SAH	40-49	142	Mean 23996, range 8975 to 40399	Mean 24587, range 10227 to 41772
SAH	50-59	177	Mean 28481, range 17200 to 50795	Mean 30760, range 17630 to 53336
SAH	60-69	107	Mean 27745, range 12640 to 57886	Mean 32348, range 14988 to 82140
SAH	70-79	17	Mean 21862, range 10953 to 32386	Mean 30538, range 14945 to 41044
SAH	80-89	2	Mean 6221, range 3901 to 3901	Mean 10165, range 4883 to 4883
Multitrauma TBI	0-9	35	Mean 2979, range 867 to 11048	Mean 2652, range 121 to 9945
Multitrauma TBI	10-19	149	Mean 3949, range 1626 to 15812	Mean 3475, range 1229 to 15748
Multitrauma TBI	20-29	206	Mean 6879, range 2135 to 17899	Mean 6198, range 1681 to 17108
Multitrauma TBI	30-39	161	Mean 10906, range 3036 to 22552	Mean 10758, range 2674 to 21427
Multitrauma TBI	40-49	129	Mean 18765, range 5995 to 46390	Mean 19197, range 6250 to 47305
Multitrauma TBI	50-59	67	Mean 23781, range 3452 to 40293	Mean 24631, range 2710 to 41007
Multitrauma TBI	60-69	46	Mean 23998, range 1072 to 43178	Mean 26801, range 2638 to 46321
Multitrauma TBI	70-79	17	Mean 36391, range 5964 to 48555	Mean 46956, range 5838 to 58264
Multitrauma TBI	80-89	4	Mean 1563276, range NA to NA	Mean 3082329, range NA to NA
Isolated TBI	0-9	43	Mean 1765, range 628 to 4466	Mean 1601, range 419 to 3760
Isolated TBI	10-19	266	Mean 3306, range 1629 to 5912	Mean 2827, range 1285 to 5431
Isolated TBI	20-29	429	Mean 4379, range 1642 to 15297	Mean 3996, range 1540 to 14091
Isolated TBI	30-39	290	Mean 7869, range 3167 to 21030	Mean 7809, range 1734 to 22090
Isolated TBI	40-49	175	Mean 12227, range 2716 to 34199	Mean 12990, range 2735 to 34403
Isolated TBI	50-59	136	Mean 13294, range 2975 to 29114	Mean 13752, range 2786 to 34687
Isolated TBI	60-69	76	Mean 13618, range 3078 to 32325	Mean 15507, range 3503 to 43547
Isolated TBI	70-79	16	Mean 23008, range 3769 to 41789	Mean 26646, range 4530 to 50791
Isolated TBI	80-89	7	Mean 2388118, range NA to NA	Mean 5592451, range NA to NA
Isolated TBI	90-	2	Mean 1312395, range NA to NA	Mean 2050255, range NA to NA
Abbreviation: TISS, Therapeutic Intervention Scoring System; LOS, Length of Stay; SAH, Subarachnoid Hemorrhage; TBI, Traumatic Brain Injury; SAPS, Simplified Acute Physiology Score				

eTable 7: Slope estimates for the relation between costSRURs and SMR across diagnostic groups for Figure 3

Diagnosis	Slope	Estimate	Type
All other diagnoses	0.053	-0.29, 95% CI (-4.09, 3.51)	$costSRUR_{TISS}$
All other diagnoses	0.038	-0.58, 95% CI (-4.45, 3.29)	$costSRUR_{LOS}$
**Neurosurgical diagnosis combined**	**-0.085**	**-1.05, 95% CI (-1.73, -0.36)**	**$costSRUR_{TISS}$**
**Neurosurgical diagnosis combined**	**-0.072**	**-1.02, 95% CI (-1.69, -0.36)**	**$costSRUR_{LOS}$**
**Non-traumatic intracranial hemorrhage**	**-0.077**	**-0.85, 95% CI (-1.63, -0.07)**	**$costSRUR_{TISS}$**
**Non-traumatic intracranial hemorrhage**	**-0.065**	**-0.92, 95% CI (-1.66, -0.19)**	**$costSRUR_{LOS}$**
SAH	-0.041	-0.66, 95% CI (-1.62, 0.30)	$costSRUR_{TISS}$
SAH	-0.037	-0.59, 95% CI (-1.51, 0.33)	$costSRUR_{LOS}$
Multitrauma TBI	0.049	-0.07, 95% CI (-0.86, 0.72)	$costSRUR_{TISS}$
Multitrauma TBI	0.051	-0.04, 95% CI (-0.80, 0.72)	$costSRUR_{LOS}$
Isolated TBI	-0.127	-0.34, 95% CI (-0.80, 0.13)	$costSRUR_{TISS}$
Isolated TBI	-0.113	-0.33, 95% CI (-0.80, 0.14)	$costSRUR_{LOS}$

Abbreviations: TISS, Therapeutic Intervention Scoring System; LOS, Length of Stay; SAH, Subarachnoid Hemorrhage; TBI, Traumatic Brain Injury; SRUR, Standardized Resource Use Rate

eTable 8: Association of ICU-related factors with costSRURs and SMR

Type	Diagnosis	Variable	Estimate (95% CI)	Model
costSRUR$_{LOS}$	All other diagnoses	FTE physicians/beds	1.0772, 95% CI (0.8692, 1.3349)	Bivariable**
costSRUR$_{LOS}$	All other diagnoses	Median SAPS-II	1.0222, 95% CI (0.9808, 1.0654)	Bivariable**
costSRUR$_{LOS}$	All other diagnoses	Organisation type (independent vs not)	0.7260, 95% CI (0.4916, 1.0721)	Bivariable**
costSRUR$_{LOS}$	All other diagnoses	SMR	0.7944, 95% CI (0.2973, 2.1229)	Bivariable**
costSRUR$_{LOS}$	All other diagnoses	Total number of beds	0.9857, 95% CI (0.9701, 1.0015)	Bivariable**
costSRUR$_{LOS}$	Isolated TBI	FTE physicians/beds	1.2195, 95% CI (0.9820, 1.5144)	Bivariable**
costSRUR$_{LOS}$	Isolated TBI	Median SAPS-II	0.9987, 95% CI (0.9553, 1.0441)	Bivariable**
**costSRUR$_{LOS}$**	**Isolated TBI**	**Organisation type (independent vs not)**	**0.6291, 95% CI (0.4280, 0.9248)**	**Bivariable****
costSRUR$_{LOS}$	Isolated TBI	SMR	0.8950, 95% CI (0.7651, 1.0470)	Bivariable**
costSRUR$_{LOS}$	Isolated TBI	Total number of beds	0.9920, 95% CI (0.9677, 1.0170)	Bivariable**
costSRUR$_{LOS}$	Multitrauma TBI	FTE physicians/beds	1.0360, 95% CI (0.8062, 1.3313)	Bivariable**
costSRUR$_{LOS}$	Multitrauma TBI	Median SAPS-II	0.9898, 95% CI (0.9451, 1.0367)	Bivariable**
**costSRUR$_{LOS}$**	**Multitrauma TBI**	**Organisation type (independent vs not)**	**0.6538, 95% CI (0.4588, 0.9318)**	**Bivariable****
costSRUR$_{LOS}$	Multitrauma TBI	SMR	0.9757, 95% CI (0.7780, 1.2238)	Bivariable**
costSRUR$_{LOS}$	Multitrauma TBI	Total number of beds	0.9862, 95% CI (0.9685, 1.0043)	Bivariable**
costSRUR$_{LOS}$	Non-traumatic intracranial hemorrhage	FTE physicians/beds	1.1519, 95% CI (0.9107, 1.4571)	Bivariable**
costSRUR$_{LOS}$	Non-traumatic intracranial hemorrhage	Median SAPS-II	1.0086, 95% CI (0.9701, 1.0487)	Bivariable**
**costSRUR$_{LOS}$**	**Non-traumatic intracranial hemorrhage**	**Organisation type (independent vs not)**	**0.6354, 95% CI (0.4395, 0.9186)**	**Bivariable****
**costSRUR$_{LOS}$**	**Non-traumatic intracranial hemorrhage**	**SMR**	**0.7850, 95% CI (0.6310, 0.9766)**	**Bivariable****
costSRUR$_{LOS}$	Non-traumatic intracranial hemorrhage	Total number of beds	0.9912, 95% CI (0.9687, 1.0142)	Bivariable**
**costSRUR$_{LOS}$**	**SAH**	**FTE physicians/beds**	**1.1746, 95% CI (1.0145, 1.3599)**	**Bivariable****
**costSRUR$_{LOS}$**	**SAH**	**Median SAPS-II**	**0.9810, 95% CI (0.9644, 0.9979)**	**Bivariable****
costSRUR$_{LOS}$	SAH	Organisation type (independent vs not)	0.8200, 95% CI (0.5417, 1.2413)	Bivariable**
costSRUR$_{LOS}$	SAH	SMR	0.8624, 95% CI (0.7087, 1.0494)	Bivariable**
costSRUR$_{LOS}$	SAH	Total number of beds	1.0027, 95% CI (0.9840, 1.0216)	Bivariable**
costSRUR$_{TISS}$	All other diagnoses	FTE physicians/beds	1.0607, 95% CI (0.8581, 1.3111)	Bivariable**
costSRUR$_{TISS}$	All other diagnoses	Median SAPS-II	1.0178, 95% CI (0.9761, 1.0612)	Bivariable**
costSRUR$_{TISS}$	All other diagnoses	Organisation type (independent vs not)	0.7158, 95% CI (0.5031, 1.0184)	Bivariable**
costSRUR$_{TISS}$	All other diagnoses	SMR	0.8648, 95% CI (0.3279, 2.2804)	Bivariable**
costSRUR$_{TISS}$	All other diagnoses	Total number of beds	0.9853, 95% CI (0.9708, 1.0000)	Bivariable**
costSRUR$_{TISS}$	Isolated TBI	FTE physicians/beds	1.2172, 95% CI (0.9844, 1.5051)	Bivariable**
costSRUR$_{TISS}$	Isolated TBI	Median SAPS-II	0.9953, 95% CI (0.9526, 1.0399)	Bivariable**

**$costSRUR_{TISS}$**	**Isolated TBI**	**Organisation type (independent vs not)**	**0.6429, 95% CI (0.4283, 0.9651)**	**Bivariable****
$costSRUR_{TISS}$	Isolated TBI	SMR	0.8970, 95% CI (0.7669, 1.0493)	Bivariable**
$costSRUR_{TISS}$	Isolated TBI	Total number of beds	0.9930, 95% CI (0.9687, 1.0180)	Bivariable**
$costSRUR_{TISS}$	Multitrauma TBI	FTE physicians/beds	1.0317, 95% CI (0.7980, 1.3337)	Bivariable**
$costSRUR_{TISS}$	Multitrauma TBI	Median SAPS-II	0.9867, 95% CI (0.9412, 1.0344)	Bivariable**
**$costSRUR_{TISS}$**	**Multitrauma TBI**	**Organisation type (independent vs not)**	**0.6588, 95% CI (0.4464, 0.9723)**	**Bivariable****
$costSRUR_{TISS}$	Multitrauma TBI	SMR	0.9693, 95% CI (0.7686, 1.2225)	Bivariable**
$costSRUR_{TISS}$	Multitrauma TBI	Total number of beds	0.9857, 95% CI (0.9677, 1.0040)	Bivariable**
$costSRUR_{TISS}$	Non-traumatic intracranial hemorrhage	FTE physicians/beds	1.1324, 95% CI (0.9035, 1.4194)	Bivariable**
$costSRUR_{TISS}$	Non-traumatic intracranial hemorrhage	Median SAPS-II	1.0030, 95% CI (0.9654, 1.0420)	Bivariable**
**$costSRUR_{TISS}$**	**Non-traumatic intracranial hemorrhage**	**Organisation type (independent vs not)**	**0.6667, 95% CI (0.4544, 0.9782)**	**Bivariable****
$costSRUR_{TISS}$	Non-traumatic intracranial hemorrhage	SMR	0.8063, 95% CI (0.6439, 1.0097)	Bivariable**
$costSRUR_{TISS}$	Non-traumatic intracranial hemorrhage	Total number of beds	0.9931, 95% CI (0.9712, 1.0155)	Bivariable**
**$costSRUR_{TISS}$**	**SAH**	**FTE physicians/beds**	**1.1757, 95% CI (1.0024, 1.3790)**	**Bivariable****
**$costSRUR_{TISS}$**	**SAH**	**Median SAPS-II**	**0.9784, 95% CI (0.9620, 0.9950)**	**Bivariable****
$costSRUR_{TISS}$	SAH	Organisation type (independent vs not)	0.8257, 95% CI (0.5283, 1.2906)	Bivariable**
$costSRUR_{TISS}$	SAH	SMR	0.8471, 95% CI (0.6893, 1.0410)	Bivariable**
$costSRUR_{TISS}$	SAH	Total number of beds	1.0030, 95% CI (0.9834, 1.0230)	Bivariable**
**SMR**	**All other diagnoses**	**FTE physicians/beds**	**0.9417, 95% CI (0.9022, 0.9828)**	**Bivariable****
SMR	All other diagnoses	Median SAPS-II	0.9929, 95% CI (0.9845, 1.0014)	Bivariable**
SMR	All other diagnoses	Organisation type (independent vs not)	0.9757, 95% CI (0.8938, 1.0650)	Bivariable**
SMR	All other diagnoses	SMR	Not estimated	Bivariable**
SMR	All other diagnoses	Total number of beds	0.9980, 95% CI (0.9954, 1.0007)	Bivariable**
SMR	Isolated TBI	FTE physicians/beds	0.9908, 95% CI (0.8100, 1.2119)	Bivariable**
SMR	Isolated TBI	Median SAPS-II	0.9899, 95% CI (0.9472, 1.0345)	Bivariable**
SMR	Isolated TBI	Organisation type (independent vs not)	1.5957, 95% CI (0.9723, 2.6188)	Bivariable**
SMR	Isolated TBI	SMR	Not estimated	Bivariable**
**SMR**	**Isolated TBI**	**Total number of beds**	**1.0281, 95% CI (1.0138, 1.0426)**	**Bivariable****
SMR	Multitrauma TBI	FTE physicians/beds	1.1740, 95% CI (0.7779, 1.7719)	Bivariable**
SMR	Multitrauma TBI	Median SAPS-II	1.0185, 95% CI (0.9586, 1.0821)	Bivariable**
SMR	Multitrauma TBI	Organisation type (independent vs not)	0.9644, 95% CI (0.5069, 1.8350)	Bivariable**
SMR	Multitrauma TBI	SMR	Not estimated	Bivariable**
SMR	Multitrauma TBI	Total number of beds	0.9909, 95% CI (0.9724, 1.0097)	Bivariable**
SMR	Non-traumatic intracranial hemorrhage	FTE physicians/beds	0.9978, 95% CI (0.8692, 1.1454)	Bivariable**
SMR	Non-traumatic intracranial hemorrhage	Median SAPS-II	0.9830, 95% CI (0.9630, 1.0033)	Bivariable**

**SMR**	**Non-traumatic intracranial hemorrhage**	**Organisation type (independent vs not)**	**1.4414, 95% CI (1.0642, 1.9522)**	**Bivariable****
SMR	Non-traumatic intracranial hemorrhage	SMR	Not estimated	Bivariable**
**SMR**	**Non-traumatic intracranial hemorrhage**	**Total number of beds**	**1.0147, 95% CI (1.0065, 1.0229)**	**Bivariable****
SMR	SAH	FTE physicians/beds	1.0355, 95% CI (0.8814, 1.2165)	Bivariable**
SMR	SAH	Median SAPS-II	1.0178, 95% CI (0.9984, 1.0375)	Bivariable**
SMR	SAH	Organisation type (independent vs not)	1.2997, 95% CI (0.8826, 1.9140)	Bivariable**
SMR	SAH	SMR	Not estimated	Bivariable**
SMR	SAH	Total number of beds	1.0048, 95% CI (0.9922, 1.0175)	Bivariable**
Statistically significant predictors (95 CI does not cross 1.0) are **bolded**<br><br>Abbreviations: TISS, Therapeutic Intervention Scoring System; LOS, Length of Stay; SAH, Subarachnoid Hemorrhage; TBI, Traumatic Brain Injury; SRUR, Standardized Resource Use Rate; SMR, Standardized Mortality Rate				

eFigure 1: The relationship between GCS score and hospital mortality across units

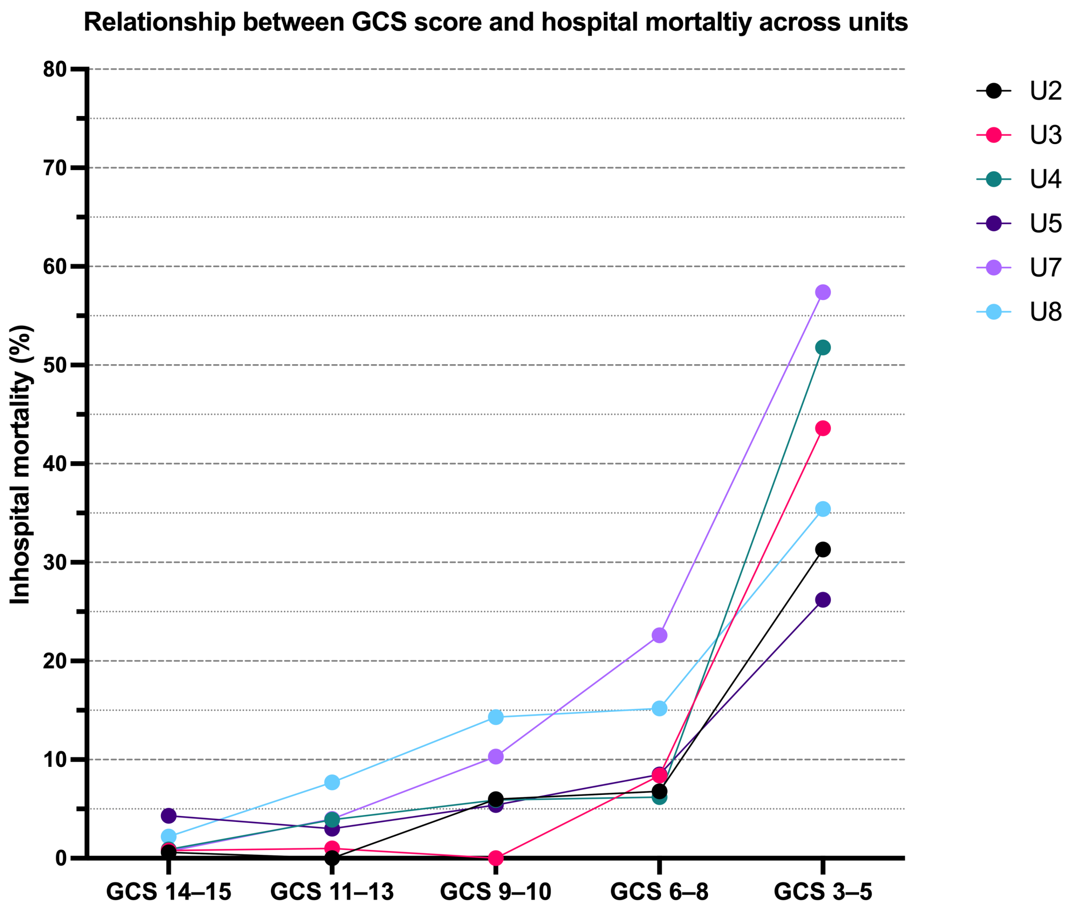

eFigure 2: Direct TISS and LOS-based cost per survivor by SAPS II strata

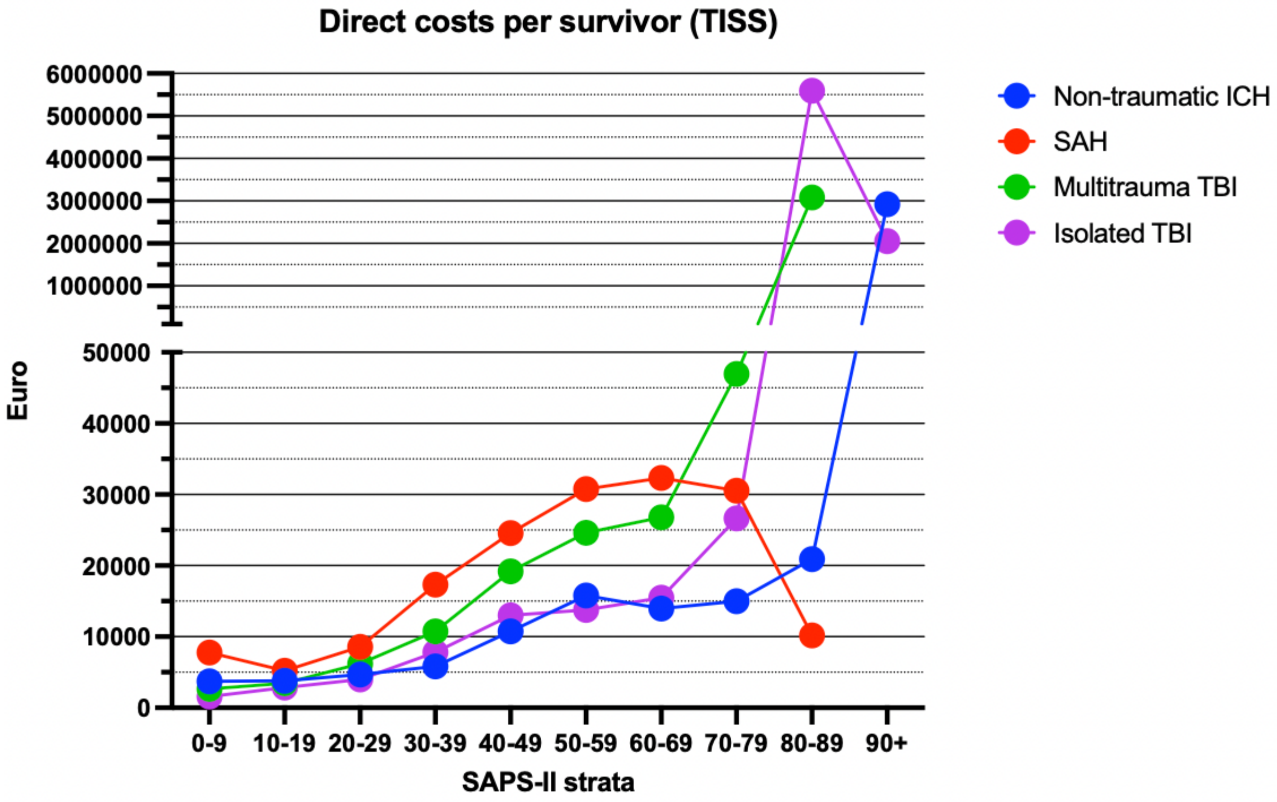

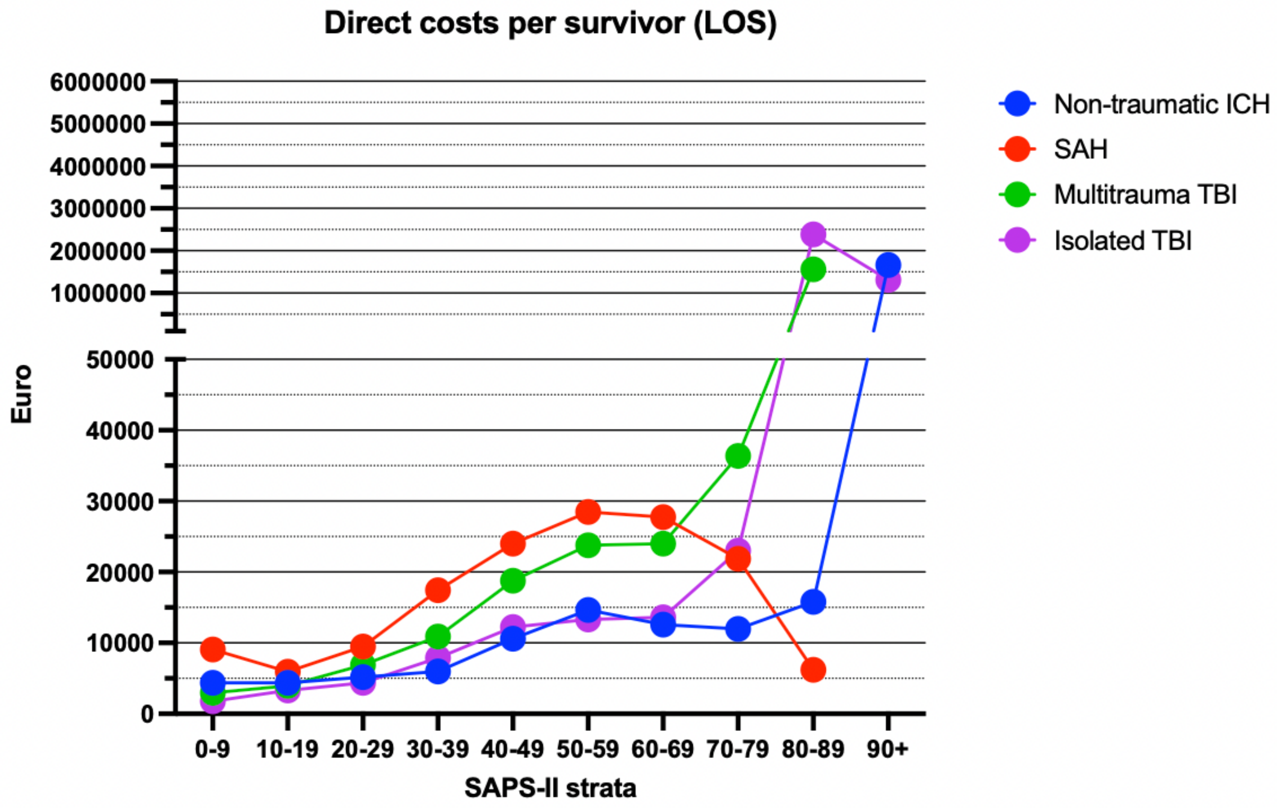

Supplement: Supplementary file 1 — Supplementary file1 (PDF 1444 KB) [file 12028_2023_1723_MOESM1_ESM.pdf]
